# Effective heterogeneous transition metal glycerolates catalysts for one-step biodiesel production from low grade non-refined *Jatropha* oil and crude aqueous bioethanol

**DOI:** 10.1038/srep23822

**Published:** 2016-03-31

**Authors:** Pak-Chung Lau, Tsz-Lung Kwong, Ka-Fu Yung

**Affiliations:** 1 Department of Applied Biology and Chemical Technology, The Hong Kong Polytechnic University, Hung Hom, Kowloon, Hong Kong; 2 Shenzhen Research Institute of The Hong Kong Polytechnic University, Shenzhen 518057, China

## Abstract

The utilization of bioethanol as the alcohol source for biodiesel production is more environmentally advantageous over methanol owing to its lower toxicity, lower flammability and its sustainable supply from renewable agricultural resources. However, as the presence of water in crude bioethanol is the critical factor limiting the biodiesel production process, the energy-intensive and costly purification of bioethanol is necessary for biodiesel application. Manganese glycerolate (MnGly) is reported the first time here as a robust heterogeneous catalyst that exhibited over 90% conversion by using aqueous ethanol containing 80 wt.% of water in the production of fatty acid ethyl ester (FAEE). The employment of 95 wt.% ethanol with respect to water could achieve 99.7% feedstock conversion in 6 hours under the optimal reaction conditions: reaction temperature (150 °C), feedstock-to-ethanol molar ratio (1:20) and catalyst loading (6 wt.%). Commercially available low grade crude bioethanol with the presence of impurities like sugars were applied which demonstrated remarkable catalytic activity in 24 hours. The high water tolerance of MnGly towards biodiesel production could eventually simplify the purification of bioethanol that consumes less energy and production cost.

Biodiesel is a promising alternative fuel to substitute petroleum-based diesel fuel in order to cope with the growing concerns on the depletion of limited fossil fuel reserves and its associated environmental problems^1^. Owing to the similarity in chemical and physical properties between biodiesel and petroleum-based diesel fuel, biodiesel can be applied to conventional diesel engines without major modifications either by direct replacement or blending with diesel fuel in an appropriate proportion^2^. Biodiesel is comprised of mono-alkyl esters of long chain fatty acids synthesized from alcohols and oil feedstock, including animal fats, vegetable oils and waste oils, through transesterification^3^,^4^. It is regarded as a renewable energy source due to its sustainable supply, carbon neutrality, biodegradability and non-toxicity^1^. However, the high price of biodiesel is the main barrier for its commercialization in the market. As the cost of feedstock accounts for 70 to 80% of total production cost of biodiesel, the application of low grade non-refined feedstock containing high free fatty acid (FFA) contaminants for biodiesel production is economically beneficial to reduce the price^5^,^6^,^7^.

The use of homogeneous strong acid and alkali catalyst is the traditional way of industrial biodiesel production. However, the high FFA contaminated non-refined feedstock causes soap formation in base-catalyzed biodiesel production^8^. The development of heterogeneous catalyst which is active and stable towards one-step simultaneous esterification and transesterification is of utmost importance to the field of renewable liquid fuel technology. Our research group has previously investigated one-step biodiesel production with methanol from low grade non-refined feedstock using basic Ca_2_Fe_2_O_5_ as catalyst. However, this catalytic system can only demonstrate limited tolerance to FFA and water^9^. ZnO nanostar has then been studied as effective catalyst towards one-step biodiesel production in which the formation of zinc oleate intermediate and zinc glycerolate side product after the reaction were reported^10^. Meanwhile, other researchers have reported the application of different metal carboxylates as heterogeneous catalyst towards biodiesel production. A series of zinc, copper (II), manganese (II), cobalt (II) and nickel (II) carboxylates were synthesized that showed significant conversions in 2 hours^11^,^12^,^13^. However, the catalytic instability due to leaching, reconstruction and transformation of the metal carboxylates after reaction was observed. Reinoso and co-workers^12^,^14^ reported the anion exchange between zinc carboxylate and fatty acid to be esterified as well as the transformation of zinc carboxylates into zinc glycerolate (ZnGly) after biodiesel production. The same research group has then reported the synthesis of ZnGly as a heterogeneous catalyst for transesterification of oil under high pressure condition which showed high robustness and tolerance to FFA and water^15^. Therefore, we are interested in exploring the catalytic activity of other transition metal glycerolates to investigate the correlation between the changes of metals ions and the final reactivity.

Methanol is currently the most common source of alcohol used in biodiesel production in the industrial sector. Despite its high environmental toxicity and flammability, the massive use of methanol is also hampered by its origin mainly from limited fossil resources^16^,^17^,^18^ which is non-renewable and is associated with the excess carbon dioxide emission. Therefore, the choice of alcohol employed is the key factor in developing a totally renewable biodiesel from sustainable source. Since ethanol can be massively obtained by alcoholic fermentation of renewable agricultural resources, it is commonly regarded as a sustainable resource and can be called as bioethanol. The utilization of bioethanol as the starting materials for biodiesel production is an excellent substitute over methanol in sustainability, production and environmental safety aspects. Also, as the molecular structures of fatty acid methyl ester (FAME) and the bioethanol derived fatty acid ethyl ester (FAEE) are similar which only differs by one methylene group, both of them are compatible with existing diesel engines^18^ which further supports that FAEE is a more logical choice for the future biodiesel.

However, research on the utilization of bioethanol towards biodiesel production is still inadequate as the application of bioethanol suffers from some technical and economical limitations. The major concern on the use of bioethanol towards biodiesel production is the presence of water as the water content in crude bioethanol from fermentation can be as high as 80%^16^. Most literatures have mentioned the presence of water as a critical factor to contribute negative influence on biodiesel production process^19^,^20^. As water induces a secondary hydrolysis of triglyceride into fatty acid, the base catalyst would react with fatty acid through saponification which hinders its catalytic activity towards biodiesel production. In order to tackle the negative effect of water on biodiesel production, crude bioethanol usually requires complicated purification and dehydration before using in production which lead to a high cost. Since ethanol-water mixture forms a minimum-boiling azeotrope at 78.2 °C on fractional distillation which ultimately yields a solution composed of only 95.6% ethanol, further dehydration process is necessary to remove the remaining water so as to generate ethanol in absolute form (>99%) with an extra cost^21^,^22^. Thus, the dehydration of bioethanol is a cost-consuming and energy-intensive process that requires 9.21–18.84 MJ/kg in making anhydrous ethanol^23^.

Moreover, as excess alcohol is required for the reversible transesterification reaction, the remaining bioethanol is normally recycled on industrial application^24^. Upon continuous consumption of ethanol in cycles of reaction, the water content in the bioethanol would be enhanced which requires energy-intensive post-purification steps *via* distillation to remove the moistures before returning to the reactor for the next cycle of reaction. Hence, the development of catalyst that is compatible with the water in ethanol is particularly important in order to save energy and purification cost of bioethanol and hence the production cost of FAEE.

In the present study, a fast and effective microwave-assisted hydrothermal synthesis of a series of transition metal glycerolates in powder form was developed. The as-prepared metal glycerolates were found to be active towards one-step FAEE production from high FFA containing crude *Jatropha* oil with bioethanol and an exceptionally high water tolerance was reported here.

## Results and Discussion

### Catalyst characterization

**Figure 1:**
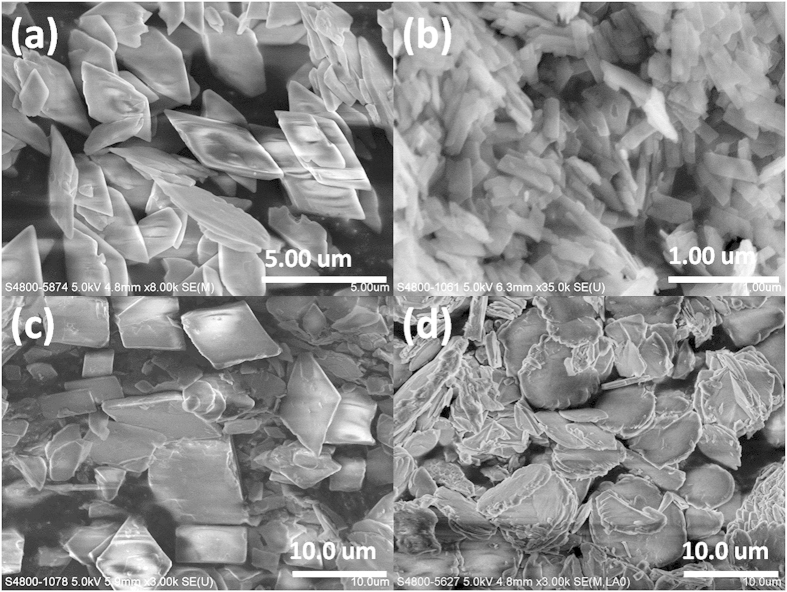
SEM micrographs of (**a**) MnGly, (**b**) FeGly, (**c**) CoGly and (**d**) ZnGly.

A series of transition metal glycerolates including CrGly, MnGly, FeGly, CoGly, NiGly, CuGly and ZnGly were explored but only MnGly, FeGly, CoGly and ZnGly can be isolated in powder form for further characterization. As the synthesis of the remaining three metal glycerolates can only lead to the formation of gel-like products, no confirmative characterization analysis can be performed to describe their identities. The morphologies of all the isolated metal glycerolates were characterized by SEM as shown in Fig. 1. MnGly, CoGly and ZnGly are found to adopt layered platelet structures while FeGly adopts a rod-shaped structure. Among all metal glycerolates prepared, MnGly shows the most regular structure which exhibits a regular parallelogram-shaped thin plate morphology with an average dimension of 3.16 ± 0.31 μm × 2.38 ± 0.25 μm. Other metal glycerolates are relatively less regular in shape with uneven sizes especially ZnGly.

**Figure 2:**
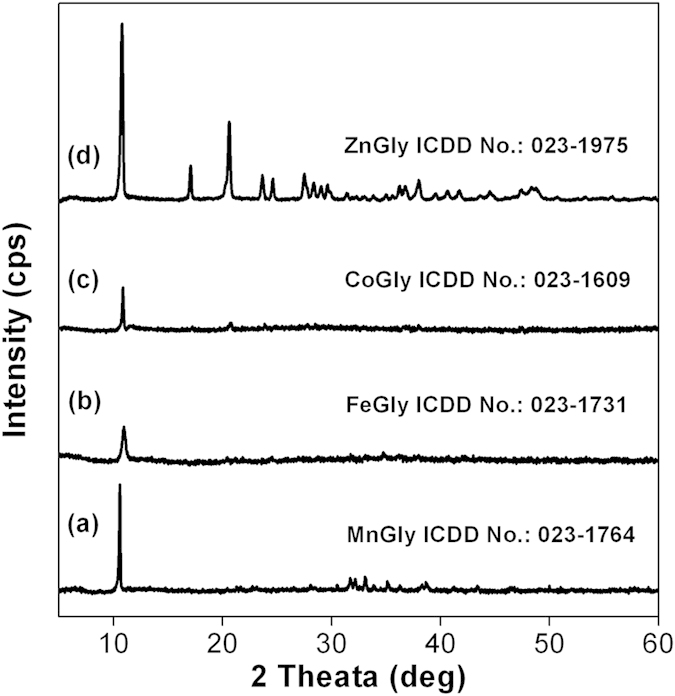
XRD spectra of (**a**) MnGly, (**b**) FeGly, (**c**) CoGly and (**d**) ZnGly.

The identity of all the isolated metal glycerolates were confirmed by XRD as depicted in Fig. 2. The characteristic diffraction peaks of the as-synthesized MnGly, FeGly, CoGly and ZnGly are matched with standard diffraction patterns according to the ICDD card No.: 023-1764, 023-1731, 023-1609 and 023-1975 respectively. The crystallinity of FeGly and CoGly are relatively lower than that of MnGly and ZnGly as observed from the smaller peak heights in the diffraction patterns of FeGly and CoGly.

**Figure 3:**
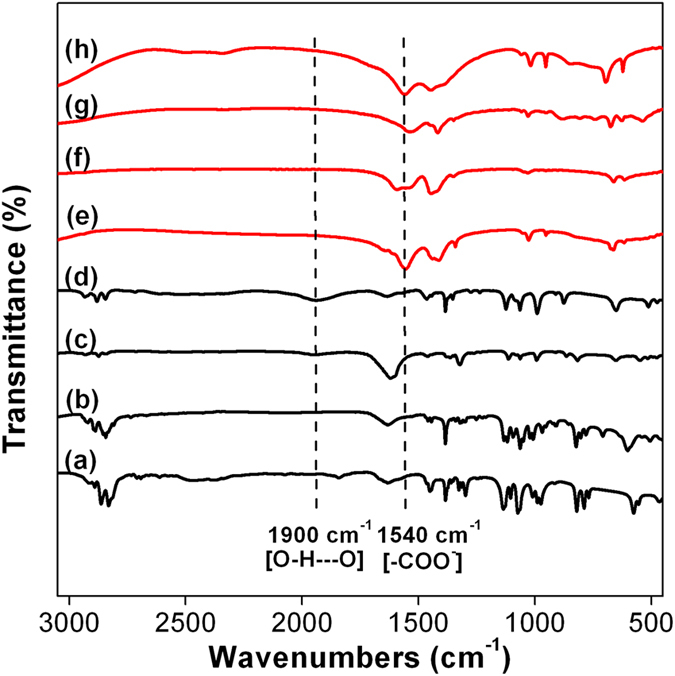
FTIR spectra of (**a**) MnGly, (**b**) FeGly, (**c**) CoGly, (**d**) ZnGly, (**e**) (CH_3_COO)_2_Mn, (**f**) (CH_3_COO)_2_Fe, (**g**) (CH_3_COO)_2_Co and (**h**) (CH_3_COO)_2_Zn.

Figure 3 shows the FTIR spectra of all the metal glycerolates and their respective metal acetate precursors. The appearance of weak signal at around 1900 cm^−1^ is ascribed to the stretching vibrations of C–O bond where the oxygen atom is involved in the O–H–O hydrogen bonding which is missing from the spectra of their respective metal acetates. On the other hand, the absence of signal at 1540 cm^−1^ due to anti-symmetric stretching of C–O bond on the carboxylate (–COO^−^) group further confirms the complete transformation of metal acetate precursors into metal glycerolates.

Based on the result of Hammett indicator analysis as shown in Table 1, the surface basic strength of MnGly, CoGly and ZnGly were found to be in the range of 6.8 < *H*_ < 7.2 which demonstrate amphoteric property on the surface of the three metal glycerolates. FeGly, however, gave a lower surface basic strength in the range of 4.8 < *H*_ < 6.8 which is slightly acidic in nature.

### Catalytic activity of metal glycerolates towards biodiesel production from crude *Jatropha* oil

The catalytic activity of different metal glycerolates towards one-step simultaneous esterification and transesterification reaction of crude *Jatropha* oil with ethanol were investigated and the results are shown in Table 1. It can be observed that MnGly exhibited the highest catalytic activity with 95.8% feedstock conversion in 4 hours. ZnGly and CoGly showed feedstock conversions of 80.0% and 50.5% respectively whereas FeGly displayed the worst performance with a conversion of 12.0%. The relatively lower catalytic performance of CoGly and FeGly could be ascribed to the low crystallinity as shown from the XRD analysis. Moreover, it is reported that amphoteric catalyst is desirable for one-step esterification and transesterification reaction due to the presence of both Lewis acid and base catalytic sites^25^. Therefore, the catalytic activities of amphoteric MnGly, CoGly and ZnGly were better than that of acidic FeGly which requires harsher reaction conditions.

On the other hand, the coordination geometry of metal glycerolates is proposed to be one of the factors affecting their catalytic activities towards FAEE production. The coordination geometry of different metal complexes varies with the type of ligands bonded and the coordination preference of metal center. Since Mn^2+^ and Zn^2+^ adopt high spin d^5^ and d^10^ electronic configuration respectively, there is only a small change in their crystal field stabilization energies between the tetrahedral and octahedral geometries. As a result, MnGly and ZnGly are believed to show no coordination preference for tetrahedral or octahedral geometries, implying that both glycerolates exhibit flexible coordination geometry to form stable transition state involving the conversion of tetrahedral to octahedral to tetrahedral state during the catalytic cycle as proposed in the transesterification mechanism^14^. However, as Fe^2+^ and Co^2+^ show d^6^ and d^7^ electronic configuration respectively that possess preferred geometry, there may be a mismatch in the transition state geometry formed. The coordination preference of metal center may cause a destabilization in the transient yield which impairs the overall catalytic activity. This may explain why MnGly and ZnGly demonstrated higher catalytic activities than FeGly and CoGly.

Structurally, Mn(II) ion is a stronger Lewis acid than the fully-filled Zn(II) ion as Mn(II) ion is more electron deficient to accept the lone pair electrons from both ethoxide anion and triglyceride. The higher catalytic performance of MnGly than that of ZnGly could be attributed to the fact that Mn(II) center of MnGly catalyst is more approachable by the reactants during the reaction. Since MnGly gave the best performance towards biodiesel production from crude *Jatropha* oil with ethanol among all metal glycerolates, MnGly was chosen for further investigation on its catalytic activity towards aqueous bioethanol.

### Catalytic activity towards simulated bioethanol of different water content

The presence of water in crude bioethanol is usually regarded as a detrimental factor towards biodiesel production^19^,^20^,^26^,^27^. The interaction between catalyst surface and water molecules is one of the major causes for the inhibition of heterogeneous catalyst as water molecule would adsorb on the catalyst surface. This would eventually block the access of reactants to the catalyst and reduce the rate of reaction^28^. Besides, the presence of surface bounded water may induce the hydrolysis of triglyceride into fatty acid which is another critical factor limiting the yield of biodiesel. As a result, the dehydration and purification of bioethanol is a crucial process for biodiesel application which is an energy-intensive and complex process with high production cost.

Therefore, the water tolerance of MnGly catalyst towards biodiesel production from crude *Jatropha* oil and simulated bioethanol of different water content was examined. A series of 95 to 20 wt.% aqueous ethanol were generated by the addition of 5 to 80 wt.% water into absolute ethanol respectively to simulate the water content in bioethanol as illustrated in Table 2. A decreasing trend in the feedstock conversion was observed from 93.8 to 50.6% when the water content in ethanol increased from 5 to 80 wt.% under the same reaction time of 5 hours. The use of 95 wt.% ethanol was commonly reported to cause a drastic decline in biodiesel yield but its application to MnGly catalytic system has only led to a slight reduction in the conversion from 99.1% to 93.8% when compared to absolute ethanol^29^. Based on the high catalytic activity of MnGly towards the FAEE synthesis using 95 wt.% ethanol as source, we extended this study to further investigate its reactivity for ethanol containing higher percentage of water in prolong time. Upon prolonging the reaction time to 10 hours, the catalyst can withstand 50 wt.% water in ethanol with feedstock conversion over 98%. Even when the water content in ethanol was increased to 80 wt.%, the conversion can still achieve 71.8%. Although longer reaction time was required to obtain higher conversion, these findings support the fact that MnGly produces FAEE that fulfills the EN 14214 standard yield requirement (at least 96.5% alkyl ester present in the biodiesel) even there is 50 wt.% of water present in the ethanol used. In literature, so far there is no other heterogeneous metal oxide based catalyst reported can achieve such high water tolerance for FAEE synthesis^17^,^30^,^31^. This could be attributed to the fact that the active sites of common base heterogeneous catalyst, such as alkaline earth metal oxides, can easily be poisoned by chemisorption of water and carbon dioxide, resulting in the inhibition of the catalysts. The exceptionally high water tolerance of MnGly towards biodiesel production could be attributed to the adsorption of oleic acid on the catalyst surface, enhancing the surface hydrophobicity. The hydrophobic catalyst surface would repel hydrophilic water molecules so as to protect the catalyst from water inhibition.

### Study of free fatty acid (FFA) content in the feedstock

**Figure 4:**
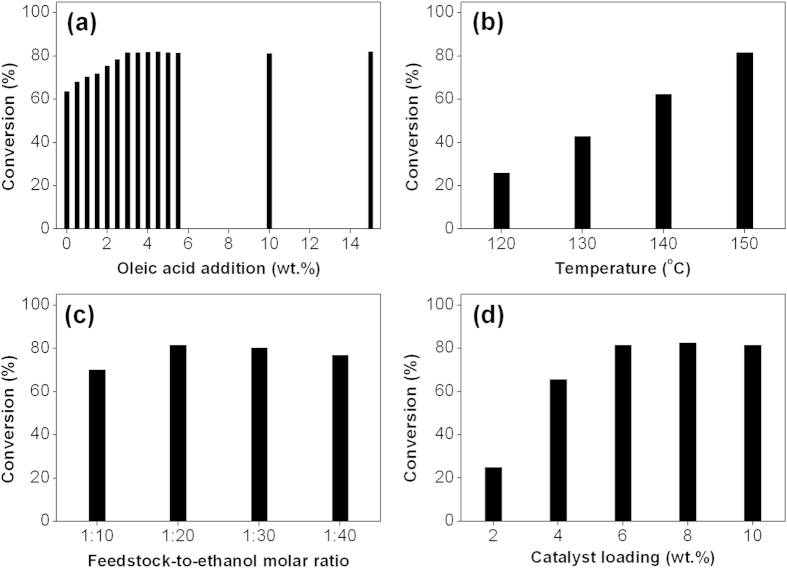
(**a**) Effect of oleic acid content on MnGly catalyzed biodiesel production using 95 wt.% ethanol. Reaction conditions: reaction temperature (150 °C), feedstock-to-ethanol molar ratio (1:20), catalyst loading (6 wt.%) and reaction time (3 h). Effect of (**b**) reaction temperature, (**c**) feedstock-to-ethanol molar ratio and (**d**) catalyst loading on MyGly catalyzed one-step simultaneous esterification and transesterification with 95 wt.% ethanol and 3 wt.% of oleic acid.

Further analysis on the effect of FFA content in feedstock was done by the addition of different weight amount of oleic acid as model of FFA into refined food grade canola oil and the result is depicted in Fig. 4a. It is observed that the feedstock conversion increased steadily from 63.6% to 81.6% when the oleic acid content increased from 0 wt.% to 3 wt.%. No further increment was observed beyond 3 wt.% of oleic acid added. The initial enhancement of feedstock conversion confirmed the existence of one-step simultaneous esterification and transesterification reaction in the presence of FFA in feedstock. As the reaction begins, FFA would adsorb on catalyst surface and esterification of FFA proceeds prior to transesterification of triglyceride. The high rate of esterification is attributed to the higher solubility of FFA in alcohol than that of triglyceride and the lower activation energy for esterification than transesterification^32^. Upon exceeding the critical point (3 wt.%), the catalyst surface is saturated with FFA so that the addition of more FFA up to 15 wt.% does not cause any improvement in the overall conversion. Therefore, the application of any non-refined feedstock containing 3 wt.% FFA or above in this catalytic system could maximize the rate of biodiesel production.

In order to investigate the effect of FFA content in feedstock on the catalytic activity of MnGly towards simulated bioethanol on the water tolerance, an artificially generated feedstock composing of refined food grade canola oil and 7.5 wt.% oleic acid (50% higher FFA content than crude *Jatropha* oil) was applied to the catalytic system to see if it will enhance or retard the conversion in the presence of more water. It was found that similar feedstock conversions were attained from all entries as that from crude *Jatropha* oil within 3% derivations as demonstrated from Table 2. It seems that once the amount of FFA present is increased to 3 wt.%, its promotion to the catalytic activity and water tolerance are plateaued.

Based on the above findings, a series of low grade non-refined feedstock of increasing acidities, waste cooking oil, crude rice bran oil, crude *Camelina* oil and crude *Jatropha* oil, were applied to the catalytic system using 95 wt.% ethanol. Their corresponding feedstock conversions were 98.5%, 99.1%, 99.4% and 99.8% respectively in 10 hours, proving the wide-range application of MnGly towards biodiesel production.

### Effect of reaction parameters

The optimization of reaction conditions in the production of biodiesel is an important stage for industrial application in order to maximize the product yield and minimize the production cost. The relationship between the catalytic activity of MnGly and the reaction parameters, including reaction temperature, feedstock-to-ethanol molar ratio and catalyst loading were evaluated. After the optimization process, the MnGly catalyzed biodiesel production with 95 wt.% ethanol was optimized at 150 °C with 1:20 feedstock-to-ethanol molar ratio under the assistance of 6 wt.% catalyst in the presence of 3 wt.% oleic acid. A feedstock conversion of 99.7% was achieved after 6 hours of reaction under the optimal reaction conditions.

The effect of reaction temperature on MnGly catalyzed biodiesel production was investigated in the range of 120 to 150 °C as depicted in Fig. 4b. Reaction temperature is found to be the most significant factor in biodiesel production process as the feedstock conversion increased significantly from 25.9 to 81.6% in the studied range. This observation could be attributed to the inhibition of mass transfer resistance in the reaction medium in case of heterogeneous catalysis. Since the addition of solid MnGly catalyst into the reaction medium generates a three phase system of feedstock-ethanol-catalyst, reaction could only be occurred at the interface of the triple phase^9^,^33^,^34^. Thus, the rate of reaction is mass transfer-controlled. The enhancement in reaction temperature would accelerate the reaction rate by reducing the viscosity of feedstock through the improvement in the mass transfer resistance. Moreover, the increase in temperature increases the number of particles having sufficient energy to overcome the activation barrier so as to increase the rate of diffusion and the chance for collision between reactants and catalysts. Hence, the optimal reaction temperature is chosen to be 150 °C.

In order to study the effect of feedstock-to-ethanol molar ratio on MnGly catalyzed biodiesel production, the reaction was investigated at 150 °C at four different levels, 1:10, 1:20, 1:30 and 1:40. It is found that the feedstock conversion increased from 70.2 to 81.6% when the feedstock-to-ethanol molar ratio was varied from 1:10 to 1:20, but decreased steadily to 77.0% at the ratio of 1:40 as shown in Fig. 4c. As transesterification reaction involves three consecutive reversible steps in converting single triglyceride and three moles of ethanol into three FAEE, an excess feedstock-to-ethanol molar ratio is desirable to shift the equilibrium to the product side^9^,^34^,^35^,^36^. However, further increase in the feedstock-to-ethanol molar ratio beyond the optimal molar ratio (1:20) lowers the concentration of feedstock in the reaction mixture which reduces the rate of reaction. In addition, the total amount of water present in 95 wt.% ethanol is higher when the feedstock-to-ethanol molar ratio increases, the chance of blockage of the active sites on the catalyst is enhanced and the catalytic activity of MnGly would be hindered. Thus, the optimal feedstock-to-ethanol molar ratio is found to be 1:20.

The effect of catalyst loading on the catalytic activity of MnGly was studied in the range of 2 to 10 wt.% at 150 °C with a feedstock-to-ethanol molar ratio of 1:20. As displayed from Fig. 4d, it demonstrates that the catalytic conversion increased significantly from 25.0% to the maximum point at 81.6% with an increase of catalyst loading from 2 to 6 wt.%. This initial improvement could be ascribed to the enhanced availability of active sites on the catalyst^9^,^34^,^37^,^38^,^39^. Furthermore, since the presence of water in the reaction medium would compete with reactants for the active sites of catalyst, the availability of more active sites would lessen the negative impact of the competing reactions. However, further increase in the catalyst loading to 10 wt.% did not show any significant enhancement on feedstock conversion as the catalytic system might be saturated with the maximum number of active sites. Therefore, the catalyst loading is optimized at 6 wt.%.

### Study of catalyst reusability

The recovery of catalyst from the reaction medium is one of major advantages of using heterogeneous catalyst in biodiesel synthesis. The used MnGly catalyst can be reactivated upon microwave irradiation with new batch of glycerol under the same catalyst preparation conditions. The catalytic performance of the reactivated MnGly catalyst was investigated under the optimized reaction conditions and the catalytic activity can be retained with over 97.5% conversion without significant deactivation for at least three cycles of reactivation.

### Application of different crude bioethanol sources towards biodiesel production

Crude bioethanol generated from fermentation of carbohydrates usually achieves roughly 20 wt.% of aqueous ethanol solution. As MnGly catalytic system has confirmed to exhibit an excellent water tolerance up to 80 wt.% in ethanol, this catalytic system can be applied to some low-cost non-refined renewable bioethanol sources. In order to evaluate the utilization of crude bioethanol on MnGly catalytic system, an artificially made aqueous ethanol sample comprising of 80 wt.% water was applied to simulate the high water containing crude bioethanol with a feedstock conversion of 91.5% after 24 hours. After that, two commercially available low grade bioethanol samples were studied under the same reaction conditions as described in Table 3. The use of singly distillated rice wine composed of 24.4 wt.% ethanol was found to have a remarkable conversion of 98.9% after 24 hours while the application of crude glutinous rice wine of 14.1 wt.% ethanol containing 10 wt.% glucose showed a conversion of 88.5%.

For comparison, 1 wt.% of NaOH was applied as homogeneous catalyst towards biodiesel production with the three bioethanol sources, a gradual decrease in feedstock conversions was observed as summarized in Table 3. Even the reaction time was prolonged to 24 hours, NaOH failed to catalyze FAEE production from crude bioethanol with feedstock conversions less than 40%. The negative impact of water on NaOH catalyzed biodiesel production could be ascribed to the occurrence of saponification^40^.

As a result, MnGly has proved to be a robust catalyst that overcomes the negative influence of water in crude bioethanol which can be applied directly towards biodiesel production. Although the utilization of crude bioethanol on MnGly catalyzed biodiesel production is an energy inefficient process that requires a longer reaction time to achieve completion, further investigation is currently undertaking to further improve the rate of reaction so as to achieve a more energy efficient production using crude bioethanol.

## Materials and Methods

### Materials

Refined food grade canola oil was purchased from a local store in Hong Kong while crude *Jatropha* oil was produced from cold-pressed oil extractor using *Jatropha* seed which was supplied by a local store in China. Manganese (II) acetate tetrahydrate ((CH_3_COO)_2_Mn·4H_2_O, 97%) was obtained from Fraco Chemical Supplies. Glycerol (C_3_H_8_O_3_, >99%), cobalt (II) acetate tetrahydrate ((CH_3_COO)_2_Co·4H_2_O, >98%) and iron (II) acetate ((CH_3_COO)_2_Fe, 95%) were purchased from Acros. Zinc acetate dihydrate ((CH_3_COO)_2_Zn·2H_2_O, 98.5%) was provided by BDH Chemical Ltd. Ethanol (C_2_H_5_OH, 99.0%) of reagent grade was supplied by ACS. Oleic acid (C_18_H_34_O_2_, 99.9%) was purchased as laboratory reagent grade from Fisher Chemical. Rice wine (24.4 wt.% ethanol) and glutinous rice wine (14.1 wt.% ethanol) were purchased from local stores in Hong Kong. Methyl yellow, methyl red, neutral red, bromothymol blue and phenolphthalein were supplied from Sigma Aldrich. Sodium hydroxide (NaOH, 96.0%) of analytical reagent was purchased from UNI-CHEM.

### Feedstock evaluation

Fatty acid composition of the feedstock used in this study were determined by Hewlett-Packard 5890 SERIES II gas chromatography equipped with a flame ionization detector (FID) and a Durabond-Wax capillary column (30 m × 0.25 mm × 0.5 μm). Both the capillary injection system and the detector system were operated at 280 °C. The column temperature was programmed from 180 to 240 °C at a rate of 2 °C min^−1^. Table 4 below shows the fatty acid composition of the feedstock. The fatty acid content and acid value of the feedstock were determined by titration based on ASTM D664 while the water content of the feedstock were evaluated by Karl Fischer titration using V20 Volumetric Karl Fischer Titrator based on ASTM D4377. Acidity, acid value and water content of the feedstock are summarized in Table 4.

### Catalyst preparation

A series of metal glycerolate catalysts, manganese (II) glycerolate (MnGly), cobalt (II) glycerolate (CoGly), iron (II) glycerolate (FeGly) and zinc glycerolate (ZnGly), were synthesized by dissolving (CH_3_COO)_2_Mn·4H_2_O (0.5 g, 2.04 mmol), (CH_3_COO)_2_Co·4H_2_O (0.5 g, 2.01 mmol), (CH_3_COO)_2_Fe (0.5 g, 2.88 mmol) and (CH_3_COO)_2_Zn·2H_2_O (0.5 g, 2.28 mmol) into glycerol (12.5 mL) respectively. The reaction mixture was placed into the pressurized microwave synthesis system (Discover SP, CEM Corporation) at 200 °C for 2 hours in which the microwave system was operated at 250 W at a fixed power mode. The resulting precipitate was centrifuged, washed with ethanol three times and finally dried at 80 °C.

### Catalyst characterization

Powder X-ray diffraction (XRD) patterns of metal glycerolates were obtained on Rigaku SmartLab X-ray diffractometer with parafocusing Bragg-Brentano geometry using CuKα radiation of wavelength 1.54056 Å with scattering angle 2θ in the range of 5° to 60°. The diffractometer was operated at 45 kV and 200 mA with step size of 0.02° and scanning speed of 5° min^−1^. The size and morphology of metal glycerolates were determined by Hitachi S-4800 field electron scanning electron microscope (SEM) which was operating at 5 kV. Fourier transform infrared (FTIR) spectrum of the catalyst using KBr pellet technique was collected in the range of 4000–400 cm^−1^ by using Nicolet Avatar 360 Fourier transform spectrometer. Hammett indicator analysis was used to elucidate the surface basicity of the catalysts in which methyl yellow (*H*_ = 3.3), methyl red (*H*_ = 4.8), neutral red (*H*_ = 6.8), bromothymol blue (*H*_ = 7.2) and phenolphthalein (*H*_ = 9.7) were used as Hammett indicators. The catalysts (5 mg) were immersed in methanolic Hammett indictor solution (1 mL, 50 μM) under ultrasonic irradiation and were allowed to stand for 1 hour to achieve equilibrium.

### Catalytic test for biodiesel production

All the catalytic reactions for biodiesel production were conducted in a stirred batch reactor containing metal glycerolate catalyst, alcohol and feedstock sample (0.46 g) with different ratios which were specified in results and discussion part. The reaction was heated with a constant stirring at 750 rpm at the corresponding reaction temperature and reaction time. The synthesized biodiesel layer was separated from catalyst by centrifugation. The feedstock conversion was analyzed by ^1^H nuclear magnetic resonance (NMR) spectroscopy on Bruker 400 MHz spectrometer using CDCl_3_ as solvent. The feedstock conversion was calculated based on the integrated ratio of the signal of –OCH_2_ on FAEE over that of the α–CH2 on triglyceride and FAEE as follows^41^,


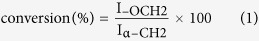


## Conclusion

The utilization of bioethanol for biodiesel production over fossil derived methanol is more favorable as the raw materials involved can be entirely renewable. A series of metal glycerolates were explored as heterogeneous catalysts towards biodiesel production in which MnGly was found to be the most promising catalyst towards aqueous ethanol and crude *Jatropha* oil. MnGly exhibited an excellent water tolerance which can withstand the presence of 80 wt.% water in ethanol for over 90% conversion with a prolonged reaction time. Overall conversion of 99.7% was achieved within 6 hours when 95 wt.% aqueous ethanol was employed under the optimal reaction conditions. Commercially available crude bioethanol with the presence of only 14.1 wt% of ethanol and 10 wt.% of glucose was also tested and confirmed to show nearly 90% conversion in 24 hours. This implication would probably lead to the simplification of complicated and energy-intensive purification steps of bioethanol on biodiesel application that makes the process more sustainable.

## Additional Information

**How to cite this article**: Lau, P.-C. *et al*. Effective heterogeneous transition metal glycerolates catalysts for one-step biodiesel production from low grade non-refined *Jatropha* oil and crude aqueous bioethanol. *Sci. Rep*. **6**, 23822; doi: 10.1038/srep23822 (2016).

## Figures and Tables

**Table 1:** Surface basicity and catalytic activity of different metal glycerolates towards transesterification of crude Jatropha oil with ethanol.

Entry	Metal glycerolate	Surface basic strength	Conversion^a^ (%)
1	MnGly	6.8 < *H_* < 7.2	95.8
2	FeGly	4.8 < *H_* < 6.8	12.0
3	CoGly	6.8 < *H_* < 7.2	50.5
4	ZnGly	6.8 < *H_* < 7.2	80.0

^a^Reaction conditions: reaction temperature (150 °C), feedstock-to-ethanol molar ratio (1:20), catalyst loading (6 wt.%) and reaction time (4 h).

**Table 2:** Catalytic activity of MnGly towards biodiesel production from simulated bioethanol of different water content.

Ethanol (wt.%)	Conversion^a^ (%)			
	Crude *Jatropha* oil		Refined canola oil with 7.5 wt.% oleic acid	
	5 h	10 h	5 h	10 h
100	99.1	–	99.9	–
95	93.8	99.8	94.4	99.9
90	86.9	99.6	87.7	99.5
85	82.3	99.3	82.1	99.5
80	80.4	99.6	79.1	99.3
75	76.4	98.8	76.6	99.6
70	72.5	99.0	73.6	99.5
60	68.9	98.3	70.1	98.5
50	65.2	98.2	68.3	98.0
40	62.0	92.5	62.6	91.1
30	57.3	86.5	60.0	87.0
20	50.6	71.7	52.6	71.8

^a^Reaction conditions: reaction temperature (150 °C), feedstock-to-ethanol molar ratio (1:20) and catalyst loading (6 wt.%).

**Table 3:** Comparison on application of different bioethanol sources towards MnGly and NaOH catalyzed biodiesel production.

Entry	Ethanol source	Conversion^a^ (%)			
		MnGly^b^		NaOH^c^	
		5 h	24 h	5 h	24 h
1	Artificially made 20 wt.% ethanol	50.5	91.5	7.5	33.0
2	Rice wine (24.4 wt.% ethanol)	57.2	98.9	7.6	38.4
3	Glutinous rice wine (14.1 wt.% ethanol)	24.4	88.5	5.8	30.6

^a^Reaction conditions: reaction temperature (150 °C), feedstock-to-ethanol molar ratio (1:20) and oleic acid loading (3 wt.%).

^b^MnGly loading (6 wt.%).

^c^NaOH loading (1 wt.%).

**Table 4:** Fatty acid composition, acidity, acid value and water content of the feedstock.

Feedstock	Fatty acid composition^a^ (%)							Acidity (wt.%)	Acid value (mg_KOH_/g)	Water content (wt.%)
	C_16:0_	C_18:0_	C_18:1_	C_18:2_	C_18:3_	C_20:1_	C_22:1_			
Refined food grade canola oil	5.00	2.68	63.06	22.82	6.44	–	–	0.11	0.22	0.13
Waste cooking oil	21.88	5.15	56.73	14.48	0.86	0.90	–	0.65	1.29	0.12
Crude rice bran oil	19.33	2.79	43.66	34.22	–	–	–	2.00	3.98	0.11
Crude *Camelina* oil	6.06	2.86	21.24	20.22	29.61	15.14	4.87	3.86	7.65	0.04
Crude *Jatropha* oil	14.55	6.95	40.82	37.68	–	–	–	4.93	9.78	0.13

^a^C_16:0_ = palmitic acid, C_18:0_ = stearic acid, C_18:1_ = oleic acid, C_18:2_ = linoleic acid, C_18:3_ = linolenic acid, C_20:1_ = gadoleic acid and C_22:1_ = erucic acid.
